# Three-dimensional multi-scale model of deformable platelets adhesion to vessel wall in blood flow

**DOI:** 10.1098/rsta.2013.0380

**Published:** 2014-08-06

**Authors:** Ziheng Wu, Zhiliang Xu, Oleg Kim, Mark Alber

**Affiliations:** 1 Department of Applied and Computational Mathematics and Statistics, University of Notre Dame, Notre Dame, IN 46556, USA; 2 Department of Medicine, Indiana University School of Medicine, Indianapolis, IN 46202, USA

**Keywords:** three-dimensional model, platelet adhesion, cell flow interaction, lattice Boltzmann, stochastic receptor–ligand model, thrombus

## Abstract

When a blood vessel ruptures or gets inflamed, the human body responds by rapidly forming a clot to restrict the loss of blood. Platelets aggregation at the injury site of the blood vessel occurring via platelet–platelet adhesion, tethering and rolling on the injured endothelium is a critical initial step in blood clot formation. A novel three-dimensional multi-scale model is introduced and used in this paper to simulate receptor-mediated adhesion of deformable platelets at the site of vascular injury under different shear rates of blood flow. The novelty of the model is based on a new approach of coupling submodels at three biological scales crucial for the early clot formation: novel hybrid cell membrane submodel to represent physiological elastic properties of a platelet, stochastic receptor–ligand binding submodel to describe cell adhesion kinetics and lattice Boltzmann submodel for simulating blood flow. The model implementation on the GPU cluster significantly improved simulation performance. Predictive model simulations revealed that platelet deformation, interactions between platelets in the vicinity of the vessel wall as well as the number of functional GPIb*α* platelet receptors played significant roles in platelet adhesion to the injury site. Variation of the number of functional GPIb*α* platelet receptors as well as changes of platelet stiffness can represent effects of specific drugs reducing or enhancing platelet activity. Therefore, predictive simulations can improve the search for new drug targets and help to make treatment of thrombosis patient-specific.

## 1. Introduction

When a blood vessel ruptures or gets inflamed, the human body responds by rapidly forming a clot to restrict the loss of blood. Blood clot (thrombi) formation is a complex biological process involving an extensive system of biochemical (coagulation) reactions, platelet hydrodynamics, platelet–platelet and platelet–blood vessel wall interactions leading to ligand–receptor adhesion bond formation and platelet activation. Platelet adhesion to the vessel wall is one of the first events associated with formation of haemostatic clots and pathological thrombi.

In this paper, a new three-dimensional multi-scale model of platelet–blood flow–vessel wall interactions combining submodels at three biological scales crucial for the early platelet aggregation is introduced and calibrated to investigate how platelet stiffness, GPIb receptor expression and platelet–platelet interaction affect platelet–wall adhesion quantified in terms of platelet pause time. We implemented a novel approach of combining a recently developed platelet hybrid membrane submodel, the subcellular element (SCE) representation of the cytoskeleton network and a continuum description of the lipid bilayer to study the very first step of blood clot formation, the rapid formation of unstable bonds which slow platelets and cause platelet-flipping and adhesion to the damaged surface. The hybrid platelet model was also coupled with the lattice Boltzmann model (LBM) of blood using the immersed boundary method (IBM) to simulate platelet motion and deformation in shear flow. The kinetics-based adhesive dynamics model was also integrated into the three-dimensional model to simulate formation and disassociation of the receptor–ligand bonds during the platelet–platelet and platelet–vessel wall interactions. Parallelized model simulations were implemented on a GPU computer cluster which speeded up simulations by a factor of 100 (table 3) in comparison with CPU implementation which allowed, for the first time, to run biologically relevant predictive simulations.

By using a novel biologically calibrated three-dimensional modelling approach, it is shown that the platelet stiffness, the number of GPIb*α* platelet functional receptors and mutual interaction between platelets can significantly alter the adherence of platelets at the site of vascular injury. Our results demonstrate how a comprehensive modelling approach coupling three biologically relevant scales can provide new insights into the biomedically important problem of early thrombus development. Variation of the number of functional GPIb*α* platelet receptors as well as changes of platelet stiffness can represent effects of specific drugs for reducing or enhancing platelet activity. This emphasizes the importance of predictive simulations as they can potentially improve the search for new drug targets and help with making treatment of thrombosis patient-specific.

Damage or alteration of a blood vessel lining can result in activation of flowing platelets and their subsequent aggregation at sites of vascular injury. The ability of platelets to tether to and translocate on injured vascular endothelium relies on the interaction between the platelet glycoprotein receptor Ib*α*(GPIb*α*) and the A1 domain of von Willebrand factor (vWF-A1) [1].

Along with biochemical activation of platelets, large shear disturbances of blood flow are one of the key factors promoting pathogenic activation of platelets and formation of thrombi. In addition, platelets flowing in a whole blood exhibit increased concentrations in the vicinity of the vessel wall, making platelet–platelet interactions more frequent near vascular surfaces. Excessive accumulation of platelets at injury sites is one of the pathological events that result in acute myocardial infarction, sudden death and ischaemic stroke. This pathological process is responsible for mortality and morbidity rates higher than for any other disease, making platelets a major target for therapeutic interventions. Thus, studying individual platelet dynamics as well as platelet–platelet interactions and platelet adhesion to a vascular or thrombus surface is of high biomedical importance and urgency.

Effects of shear flow on accumulation of platelets on various surfaces have been extensively studied in *in vitro* and *in vivo* experiments [1–7]. However, there is a limited amount of available experimental data on an individual platelet dynamics in the vicinity of the vascular surface as well as platelet–surface attachment. There is also a lack of experimental data demonstrating how platelet–surface attachment is affected by mechanical properties of a platelet as well as by platelet receptor–ligand kinetics. Better understanding of platelet aggregation requires study of the interplay among biochemical, mechanical and hydrodynamic processes occurring at different scales, including a nanometre scale (receptor–ligand kinetics), a micrometre scale (cellular level) and a millimetre scale (early platelet aggregate). Multiple characteristic scales make it difficult to experimentally discern effects of different processes involved in platelet–surface attachment and overall thrombus growth dynamics. Meanwhile, a multi-scale modelling approach can provide a useful predictive tool to aid in elucidating mechanisms of platelet–wall attachment and aggregation.

Several multi-scale models attempting to couple large numbers of submodels at different scales have been developed (see, among others, for reviews [8,9]). These models implemented simplified submodels in order to make simulations less computationally expensive. It is extremely difficult at this time, if not impossible, to validate predictions of multi-scale models attempting to combine submodels at all scales representing processes of blood clot formation using existing experimental data. In addition, most experimental data are currently available at the molecular level and individual platelet level. Therefore, it is important to develop detailed multi-scale models coupling two or three scales and considering only a few processes at a time. Such models when properly calibrated with available experimental data can provide useful predictive tools aiding in designing new experiments, drug design and planning new patient-specific therapeutic strategies.

Several computational models have been developed to characterize platelet and other types of blood cell motion and adhesion dynamics under hydrodynamic shear flow at cell and receptor levels (see [8,9] for a review). Analytical solutions for forces and torques exerted on a platelet treated as a rigid object in the Stokes regime in a two-dimensional case were obtained in [1,10] and compared with the data obtained using an image analysis algorithm for tracking the motion of platelets before, during and after contact with the surface. Kinetic properties of the receptor–ligand adhesion bonds, GPIb*α*–vWF, were quantified in [1,4] using Monte Carlo simulations and pause time analysis of transient capture/release events. This approach provided association and disassociation rate constants *k*_on_ and *k*_off_, depending on the shear rate of the blood flow.

Experimental study in [11] showed that platelets have viscoelastic properties and the elastic moduli in the range of 1–50 kPa. Large deformation occurred when platelets were suspended in the shear flow [12]. To account for the elastic and viscoelastic properties of cells, a number of methods accounting for cell structural properties have been developed [13,14]. The SCE model introduced in Sandersius & Newman [13] represented each cell by a collection of elastically coupled SCEs, interacting with each other via short-range potentials. Sweet *et al.* [15] and Xu *et al.* [14] presented a three-dimensional modelling approach in which cells, modelled by SCEs, were coupled with fluid flow and substrate models by using the Langevin equation.

The fluid–structure interaction approach is an essential part of the model. Previously, the IBM introduced by Peskin [16] to investigate blood flow in the human heart has been applied to many other fluid–structure interaction problems, including platelet aggregation [17] and deformation of red blood cells [18]. Skorczewski *et al.* [19] developed a two-dimensional model, using a lattice Boltzmann IBM to investigate the motion of platelets near a vessel wall and close to an intravascular thrombus, in which they modelled the platelets as rigid bodies, whereas the red blood cells were represented as deformable bodies.

The results of the predictive simulations of the three-dimensional model introduced in this paper revealed that the platelet pause time strongly depends on the stiffness of the platelet as well as on the number of expressed GPIb membrane functional receptors. Additionally, we demonstrated that the platelet–platelet interaction near the surface of the vessel wall could significantly decrease the platelet paused time, and thus decrease the rate of platelet attachment to the injury site.

This paper is organized as follows. It starts with the Biological Background section. Then the Methodological Innovation is described in detail, including description of submodel at each of three space scales and of the coupling approach. This is followed by the Results section, which includes model validation and description of the predictive simulation. Biological relevance of the predictive simulations is discussed in the Discussion section. GPU implementation of the three-dimensional model is described in appendix A.

## 2. Biological background

The mechanism by which platelets bind to a damaged blood vessel wall is similar to that of leucocyte binding to activated endothelium [20], and requires two binding steps. The first step is the rapid formation of unstable catch–slip bonds which slow platelets and cause platelet-flipping along the damaged surface. (Counterintuitively, the dissociation rate first decreases with increasing force until reaching a threshold.) This is mediated by the platelet receptor component, GPIb*α*, forming transient bonds with the vWF exposed at the injury site. Rapid association and dissociation kinetics of the bonds result in transient tethering and subsequent flipping (or rolling) and pausing of platelets on the vessel surface [1,21]. Then, stable bonds slowly form between platelet receptors and ligands (often integrin *α*IIb*β*3 binding with vWF or fibrinogen) bound to the damaged wall or the surface of the thrombus resulting in strong adhesion, initiating transmembrane and, subsequently, intracellular signalling. As the blood clot grows, platelet–platelet interaction becomes one of the major factors determining clot growth rate and integrity as platelets expose GPIIbIIIa receptors which permit platelet–platelet adhesion via fibrinogen. Adhesion of platelets to the injured surface is also affected by shear rates of the flow. At high shear, platelet integrin *α*2*β*1 and GPVI receptors are not sufficient to initiate binding to collagen, and binding of the GPIb*α* receptor to vWF immobilized on collagen becomes essential in platelet adhesion.

The stiffness of the platelet not only determines the shape and morphology of the clot, but also affects clot mechanical properties, as platelet stiffness determines cell shape when exposed to various flow conditions and contact interaction with other cells and blood vessel wall. This will affect the number of receptor–ligand pairs in platelet–platelet and platelet–substrate interactions. Platelet stiffness is also an important property reflecting platelet functioning, because it reorganizes its structure during activation or as a response to physiological or pathological conditions.

To date, to the best of our knowledge, cumulative effects of platelet stiffness, different levels of expression of GPIb*α* receptors and platelet–platelet interaction impacting the strength of platelet–substrate binding have not been systematically investigated. Our model provides a unique means for quantitatively understanding these effects, which are critical for improving our knowledge about the initial stage of the blood clot formation.

## 3. Methodological innovation of the three-dimensional modelling approach

**Figure 1.**
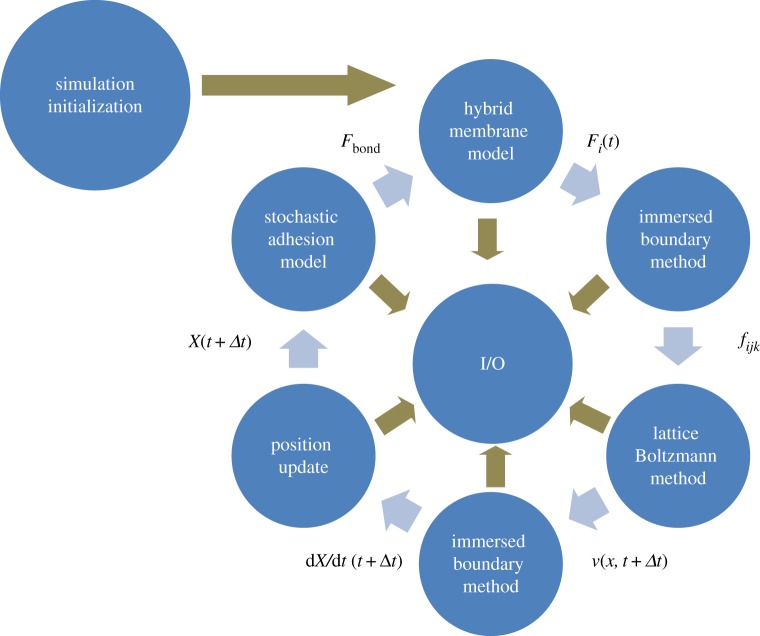
Flow chart of the simulation algorithm. Using *F*_*i*_(*t*) hybrid membrane model, the forces *F*_*i*_(*t*) acting on cell elements were calculated. The forces *f*_*ijk*_ acting on fluid nodes were spread from *F*_*i*_(*t*) by the immersed boundary method. The velocity field *v*(*x*,*t*+Δ*t*) of fluid was obtained by the lattice Boltzmann method. The velocities of cell elements d*X*/d*t*(*t*+Δ*t*) were determined by immersed boundary interpolation. The cell elements positions were updated based on the velocities. Finally, the stochastic adhesion model was used to determine the force *F*_bond_ acting on receptor–ligand bonds that bind platelets to vessel walls. (Online version in colour.)

The novelty of the three-dimensional model lies in developing a novel membrane submodel as well as in new approaches of coupling submodels of biological processes at three spatial scales (figure 1) which are crucial to early blood clot formation. At the subcellular scale (nanoscale), a kinetics-based stochastic dynamic adhesion submodel (DAM) is used to simulate vWF–GPIb*α* binding and GPIb*α*–vWF–GPIb*α* binding, in which individual vWF and GPIb*α* molecules are represented as elastic springs (table 1). This is justified by the fact that these receptor–ligand bindings are probabilistic in nature [1]. Moreover, individual filaments in the cytoskeleton network of the platelet membrane are treated as coarse-grained harmonic springs. At the cellular scale, a novel continuum description of the lipid bilayer of the cell membrane is used. We developed this new platelet membrane model to study effects of membrane stiffness on cell–substrate interaction, which was shown to strongly affect platelet–injury site adhesion. (See also §4*c*(i) for model prediction.) The subcellular scale and the cellular scale components are integrated by distributing GPIb*α* receptors at the vertices of the cytoskeleton network and by superimposing the cytoskeleton network and the lipid bilayer. At the macroscale, the dynamics of the fluid flow is represented using the LBM to facilitate parallelizing the simulation code on GPUs. The platelet model is coupled with the LBM using the IBM. (The coupling and data flow between all the submodels are demonstrated in figure 1.) We calibrate and validate this three-dimensional model by comparing simulations at different scales with either theoretical results or available experimental data at these scales. Specifically, the platelet model coupling with the LBM was validated using theoretical results and previous simulation results (see also §4*a*), whereas the platelet–substrate adhesion simulations were compared with experimental data to calibrate the DAM under different flow conditions (see also §4*b*).

**Table 1.** Biological processes and submodels at different scales.

scales	processes	submodels	coupling
<0.1 μm subcellular-level nanoscale	ligand–receptor interactions	stochastic dynamic adhesion model	1. Nano–micro scales: coupled by explicitly modelling receptors on platelet membrane nodes
approximately 1 μm cellular-level microscale	individual platelet deforming, flipping and adhering to vessel wall	hybrid membrane model	2. Micro–macro scales: coupled through immersed boundary method
>10 μm macroscale	blood flow and its Interaction with platelet	lattice Boltzmann method	

At each time step of simulation, the hybrid membrane model is first used to calculate forces acting on the nodes of the Lagrangian mesh representing platelet geometry, such as bond forces resulting from stretching or compression of the cytoskeleton network, bending forces resulting from deformation of the lipid bilayer and attraction/repulsion between platelets and the environment owing to formed ligand–receptor bonds. This is followed by coupled LBM and IBM to update fluid flow and position of platelets. Finally, the MC computations of platelet adhesion to a surface expressing vWFs are performed to break the already formed bonds and to generate new bonds from unbound GPIb*α* and vWF.

We note that this is the first time that a detailed platelet membrane model has been developed and implemented on GPUs for studying cell–flow, cell–cell and cell–substrate interactions. Because of the speed-up gained by GPU implementation, we are able to investigate effects of these interactions and cell mechanics on platelet dynamics in a timely manner. Additionally, this model can be directly used for modelling any biological cells with membrane structures similar to these of eukaryotic cells. Here, we describe in detail individual submodels and explain how they are coupled.

### (a) Platelet membrane submodel

**Figure 2.**
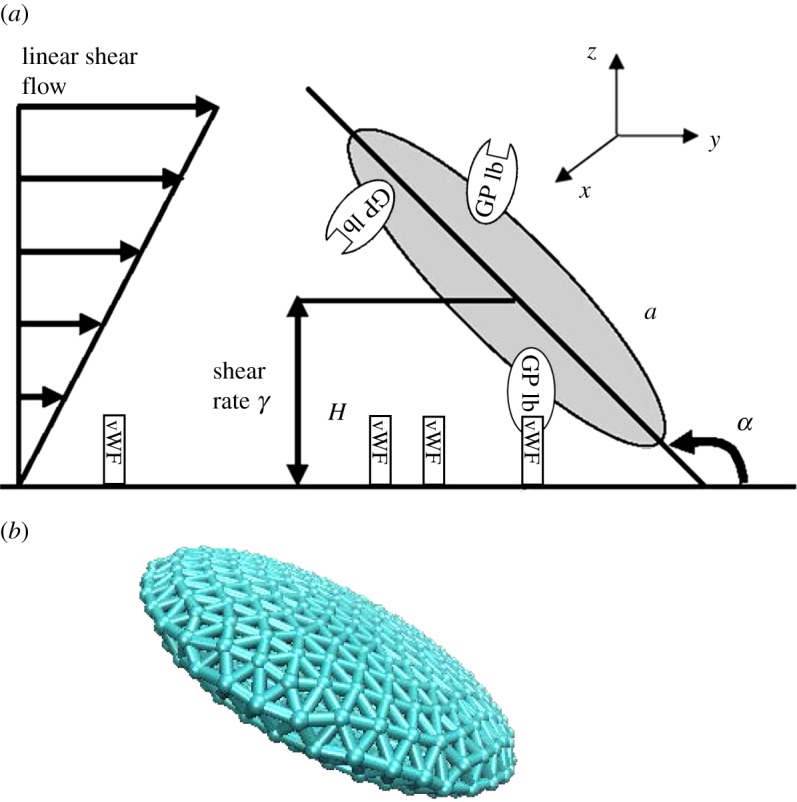
(*a*) Schematic diagram showing one platelet translating and rotating in shear flow near an infinite plane wall. (*b*) Structure of a platelet consisting of 958 SCEs. The major radius, *a*, and centroid height, *H*, are defined as is the coordinate system and flow direction. One platelet is represented by a collection of elastically linked SCEs, interacting with one another via a spring-like elastic force. The GPIb*α* receptors are randomly uniform distributed on the platelet membrane, and vWF ligands are distributed on the wall. (Online version in colour.)

We simulate the motion of platelets in a three-dimensional region bounded by an infinite flat plane at *z*=0 (see figure 2*a* for example). A platelet has initial shape defined by *x*^2^/*a*^2^+*y*^2^/*a*^2^+*z*^2^/(*λa*)^2^=1, where *a*=1 μm is the approximate particle radius and λ=0.25 is the aspect ratio [22,23]. The Reynolds number of this system is *Re*=*γρa*^2^/*μ*=*O*(10^−3^), where *γ*=300 and 400 s^−1^ are the shear rates used in experiments [1], *a*=1 μm is the particle radius, *ρ*=1.0239 g cm^−3^ is the density of blood plasma and *μ*=1.2 cP is the viscosity of blood plasma [24].

The platelet membrane, which is similar to the membrane of a red blood cell, is also assumed to consists of a lipid bilayer and an attached cytoskeleton. Following ideas from [16,25], the platelet membrane surface geometry is represented by a triangular mesh consisting of a collection of *N* (*N*=958 in our simulation) points {***X***_*i*_,*i*∈1…*N*} (figure 2*b*). The connected edges of the mesh are used to model the cytoskeleton network of the platelet membrane, and the triangulated mesh surface represents the lipid bilayer of the cell membrane, where the cytoskeleton attaches to. The mesh points represent coarse-grained actin vertices and each edge of the mesh represents a coarse-grained filament. The Helmholtz free energy of the membrane is defined as
3.1
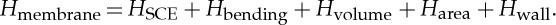

Here, *H*_SCE_ is the in-plane energy of the cytoskeleton network; *H*_bending_ is the bending energy representing the bending resistance of the lipid bilayer; *H*_volume_ and *H*_area_ are volume and area conservation constraints, respectively, and *H*_wall_ represents the energy relating to interactions due to ligand–receptor binding (explained in detail in §3*c*).

We use a harmonic ‘spring’ model to simulate the elasticity of the edge connecting mesh points *i* and *j*, which mimics a coarse-grained filament. The associated potential energy functions for points *i* and *j* are
3.2
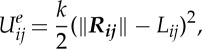

where *L*_*ij*_ is the rest length, ***R***_*ij*_=***X***_*j*_−***X***_*i*_ the position vector difference for points *i* and *j*, respectively, and *k*=*E*Δ*x*/5 the coefficient that defines the spring ‘stiffness’ [26] for elastic modulus *E*=25 kPa [11] and Δ*x*=0.1 μm unit link length of the spring. The total potential energy for the cytoskeleton network is 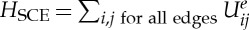. The corresponding force vector acting on point *i* by point *j* is
3.3
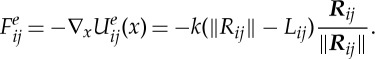

The area and volume conservation constraints, which account for area incompressibility of the lipid bilayer and incompressibility of the inner cytosol, respectively, are expressed as
3.4
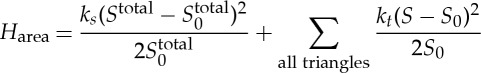

and
3.5
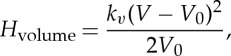

where *k*_*s*_, *k*_*t*_ and *k*_*v*_ are the global area, local area and volume constraint coefficients, respectively. The terms *S*^total^ and *V* denote the surface area and volume for the whole platelet, whereas *S*_0_, 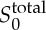 and *V*_0_ are the individual triangle mesh area, the total membrane area and the volume for unstressed platelet, respectively.

We adopt the energetic variational approach developed in [27] to represent the lipid bilayer of the cell membrane. Let *Σ*∈*R*^3^ be a smooth, closed surface representing the lipid bilayer of the platelet. The bending energy of the lipid bilayer is defined as [27]
3.6
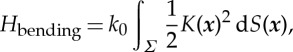

where *K*(*x*)=1/2(*κ*_1_(***x***)+*κ*_2_(***x***)) is the mean curvature, and *κ*_1_(***x***), *κ*_2_(***x***) are the principle curvatures at the point *x*. We follow the finite-element method in [28] to calculate *κ*_1_(***x***) and *κ*_2_(***x***). Briefly, let *u*(*ξ*,*η*) be a function defined over a triangle of the surface mesh representing the lipid bilayer and approximated as
3.7
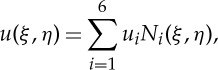

where *ξ*, *η* are the local parametric coordinates, *u*_*i*_ is the value of *u* at node *i* and *N*_*i*_(*ξ*,*η*) are the basis functions for a quadratic six-node triangular finite element. To evaluate the membrane curvature tensor ***κ***, one needs to calculate the left Cauchy–Green strain tensor, which is determined from the surface deformation gradient tensor, ***A***. For each triangular element, the surface deformation gradient tensors at the element nodes are obtained by solving the following system of equations
3.8
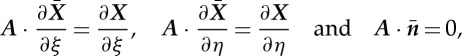

at each node of the element, and ***X***, 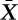 are its positions in the unstressed state and after deformation at time *t*, respectively. 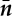 is the unit normal vector to the undeformed membranes. To evaluate the curvature tensor *κ* at a point, one needs to solve
3.9
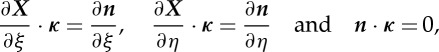

at each element node and then average over the elements sharing that node, and *n* is the unit normal vector to the deformed membranes. The mean curvature is given by
3.10
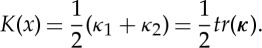

The normal component of the elastic force-associated bending energy (3.6) is obtained by taking variational derivative and is given by ***F***_bend_=(Δ_*Σ*_*K*+2*K*^3^)***n***.

Thus, nodal forces *F*_*i*_ are derived from the total energy as follows
3.11
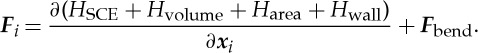

Computation of ∂(*H*_wall_)/∂***x***_*i*_ is explained in §3*c*.

### (b) Platelet stochastic dynamic adhesion submodel

The kinetics-based stochastic DAM based on ideas of the Dembo model [29,30] is used to simulate the GPIb*α* of unactivated platelet binding to immobilized vWF on the vessel–wall or platelet–platelet adhesion through forming GPIb*α*–vWF–GPIb*α* bonds, in which vWF was originally in plasma. Here, we provide details of the model for GPIb*α*–(immobilized) vWF bond formation; modelling of GPIb*α*–vWF-GPIb*α* is treated similarly. Each platelet has approximately 10 688 GPIb*α* receptors distributed uniformly on its membrane surface, to achieve a surface density of approximately 1500 receptors μm^−2^ [31]. In our model, 5344 receptor point locations on the platelet surface are randomly distributed on the platelet membrane mesh, with each point location representing two GPIb*α* receptors, because there are two GPIb*α* receptors existing on each GPV molecule [32]. On the bottom plane of the simulation domain, *z*=0, immobilized vWFs are uniformly distributed, resulting in a vWF density of 25 μm^−2^, which is consistent with experimental conditions in Doggett *et al.* [1].

The following rules are used for governing the GPIb*α*–vWF binding [33]. (i) Two vWF molecules cannot bind to the same receptor nodes for reasons of steric blocking, and (ii) receptors from a maximum of four receptor nodes present on a platelet surface can bind a vWF molecule.

In our stochastic DAM, when an unbound GPIb*α* and an unbound vWF are separated by less than the length of a GPIb*α*–vWF bond of 128 nm [34,35], a test for forming a bond is performed. Next, the formed bonds are tested for breakage. A GPIb*α*–vWF bond is modelled as a linear spring.

Probabilities of GPIb*α*–vWF bond formation and dissociation are calculated using *P*_f_ (probability of forward reaction) and *P*_r_ (probability of reverse reaction) described in [36]: 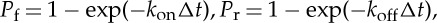 where *k*_off_ and *k*_on_ are given in s^−1^ units and Δ*t* is the simulation timestep. The reverse rate constant is calculated using the Bell model for the force-dependent dissociation rate of weak non-covalent bonds
3.12
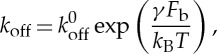

where *k*_off_(*F*_b_) is the bond dissociation rate, *k*^0^_off_ is the unstressed off-rate, *γ* is the reactive compliance, *F*_b_ is the applied force on the bond and *k*_B_*T* is the product of Boltzmann constant and temperature. The dependence of bond formation rate constant *k*_on_ on the deviation bond length is described by [29,33] as
3.13
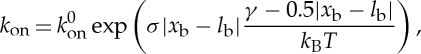

where *k*^0^_on_ is the intrinsic cross-linking formation rate constant, *σ* is the spring constant, *l*_b_ is the equilibrium bond length, *x*_b_ is the distance spanning the endpoints of the GPIb*α* receptor on the platelet surface and the vWF-A1 binding site.

The adhesion force of the GPIb*α*–vWF bond located at *i*th node of the cell membrane is calculated using a spring model as follows
3.14
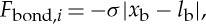

where *σ* is the spring constant, *l*_b_ is the equilibrium bond length, *x*_b_ is the distance spanning the endpoints of the GPIb*α* receptor on the platelet surface and the vWF-A1 binding site. Table 2 lists values of parameters of the DAM used in simulations.

**Table 2.** Values of physical parameters used in simulations.

parameters	definition	value	references
*a*	platelet radius	1.0 μm	[23]
λ	platelet aspect ratio	0.25	[22]
*Υ*	flow shear rate	300 and 400 s^−1^	[1]
*ρ*	blood plasma density	1.0239 g cm^−3^	[24]
*μ*	blood plasma viscosity	1.2 cP	[24]
*E*	platelet elastic modulus	25 kPa	[11]
*l*_0_	average length of initial spring length	75 nm	
*k*_*s*_	global area constraint coefficient	6000 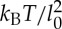	[25]
*k*_*t*_	local area constraint coefficient	6000 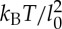	[25]
*k*_v_	volume constraint coefficient	6000 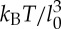	[25]
*k*_0_	bending modulus	200 *k*_B_*T*	[27]
*T*	temperature	300 K	
*k*^0^_on_	intrinsic cross-linking formation rate	10^−5^ s^−1^	[1]
*k*^0^_off_	unstressed disassociation rate	3.45 s^−1^	[1]

### (c) Lattice Boltzmann method for simulating blood flow

**Figure 3.**
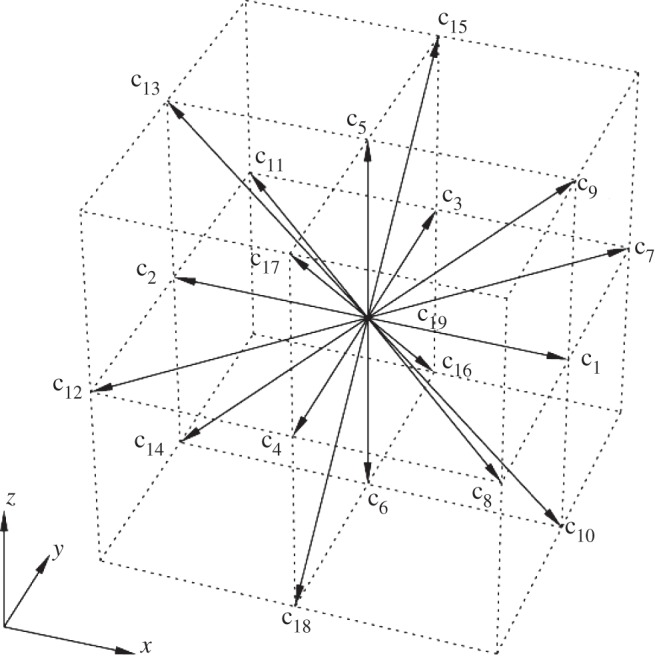
Lattice Boltzmann D3Q19 (three-dimensional and 19 velocities) model. The lattice vectors *c*_*i*_ represent the velocities of the particle moving from the centre grid point to its neighbour grid point.

The LBM uses purely localized fluid particle evolution and relaxation, which in turn facilitates parallelization in computer implementation. The LBM decomposes the fluid domain into structured lattice nodes and operates on the lattice. The fluid is modelled as a group of fluid particles that are only allowed to move between lattice nodes or remain at rest. The composition of the lattice nodes depends on the chosen lattice model. In this paper, we used the three-dimensional model of a cubic lattice (16×64×16 μm with spacing *h*=0.2 μm) with 19 discrete velocity directions (model D3Q19, as shown in figure 3). The LBM solves the Boltzmann equation describing the dynamics of fluid from a microscopic point of view: in fluid, particles with velocities *v*_*i*_ collide with certain probability and exchange momentum. The collisions are assumed to be ideal, that is the total momentum and energy is conserved during the collisions. The Boltzmann equation describes the probability *f*(*x*,*v*,*t*) of finding a particle with velocity *v* at a position *x* and at time *t* evolving with time
3.15
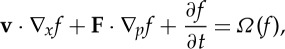

where *F* denotes an external body force, ∇_*x*,*p*_ is the gradient in position and momentum space and *Ω*(*f*) denotes the collision operator which is chosen as a relaxation of *f* with a characteristic time *τ* to the equilibrium distribution *f*^(eq)^(**v**,*ρ*):
3.16
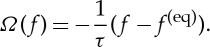

The equilibrium distribution function depends on the local density *ρ*(***x***,*t*) and the velocity field ***v***(***x***,*t*). In the D3Q19 lattice model, 19 values *f*_*i*_(**x**,*t*) are stored at each lattice site assigned to a lattice vector ***c***_*i*_. The local density at a lattice point is obtained by summing all *f*_*i*_,
3.17
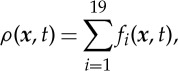

and the streaming velocity is given by
3.18
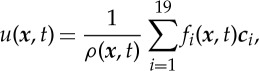

where ***c***_*i*_=*h*/Δ*t* is the lattice speed associated with the *i*th direction and Δ*t* is the time step of our simulation.

Using a Chapman Enskog expansion, Guo *et al.* [37] showed that the following lattice Boltzmann equations give a second-order accurate ***v***, the Navier–Stokes velocity in the presence of a spatially varying, time-dependent force
3.19
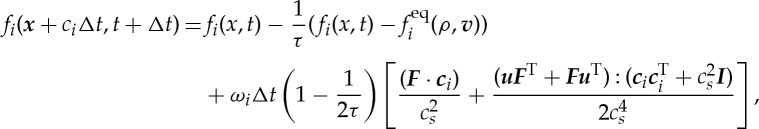

where ***u*** is a streaming velocity defined in equation (3.18), ***v***=***u***+***F***Δ*t*/2*ρ*, and
3.20
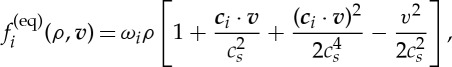

with the lattice speed of sound 
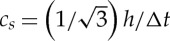
 for the D3Q19 lattice and the lattice weights
3.21
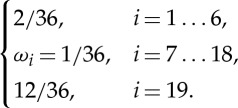

The pressure 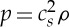 turns out to be proportional to the density and the dynamic shear viscosity is given by
3.22
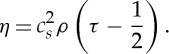



To ensure convergence and stability of LBM, we follow the method in [38] to choose our parameters. Spacing *h*=0.2 μm was determined by our simulated fluid domain and memory size of the GPU card. Time step Δ*t* was determined from the equation [38]: Δ*t*=(*τ*−0.5)*h*^2^/(3*υ*), where *υ*=*μ*/*ρ* is kinetic viscosity, *μ* and *ρ* are fluid viscosity and density as defined in table 2. Generally speaking, a larger value of *τ* leads to a more stable LBM simulation, and *τ* must be greater than 0.5. We set *τ*=1.379 s in our model, such that Δ*t*=10^−8^ s.

Periodic boundary conditions in *x*–*z* and *y*–*z* boundary planes (*y*=0, *y*=64 μm, *x*=0 and *x*=16 μm), are realized by propagating the *f*_*i*_ from the computational domain on the one boundary to the boundary on the opposite side of the domain. In the *x*–*y* boundary planes, we used the onsite velocity boundary conditions proposed by Hecht & Harting [39]. For instance, in the *x*–*y* boundary plane *z*=0, *f*_*i*_ (*i*=1, 2, 3, 4, 6, 7, 8, 10, 11, 12, 14, 16, 18, 19) can be obtained from the streaming step, but *f*_*i*_ (*i*=5, 9, 13, 15, 17) are undetermined. Following the methods of Hecht & Harting [39], we obtain
3.23
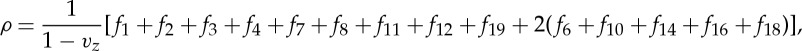

3.24
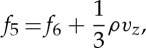

3.25
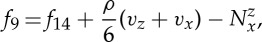

3.26
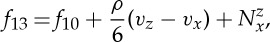

3.27
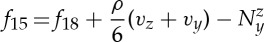

3.28
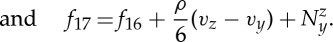

Here, *v*_*x*_, *v*_*y*_ and *v*_*z*_ are boundary velocities in *x*-, *y*- and *z*-directions, 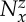 and 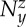 the transverse momentum corrections on the *z*-boundary for distributions propagating in *x*- and *y*-directions, respectively
3.29
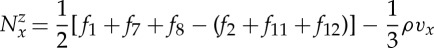

and
3.30
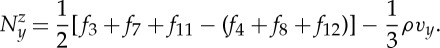



### (d) Coupling platelet, dynamic adhesion and flow submodels

#### (i) Coupling platelet and dynamic adhesion submodels

As described in §3*c*, GPIb*α* receptors are randomly distributed on the cell membrane. In each step of the simulation, forming and breaking a GPIb*α*–vWF bond is updated using the DAM. When a formed bond is either stretched or compressed, the bond deformation force is computed using equation (3.14). This force is exerted on the cell membrane at the place where the GPIb*α* receptor of the bond is located. When only the platelet membrane and vessel wall interaction is considered, the term *H*_wall_ of equation (3.1) represents the energy associated with these interactions. In particular, ∂(*H*_wall_)/∂***x***_*i*_ corresponds to the sum of following two forces (i) adhesion forces caused by GPIb*α*–vWF bond and (ii) short-range repulsive forces accounting for contact of vessel wall. The short-range repulsive force is given by an empirical relationship: *F*_rep_=*F*_0_(*τ*e^−*τε*^)/(1−e^−*τε*^), where *F*_0_=500 p Nm, *τ*=2000 μm^−1^ and *ε* is the separation distance between platelet membrane and vessel wall [29]. Thus, ∂*H*_wall_/∂***x***_*i*_ term in equation (3.11) is defined as

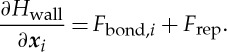



#### (ii) Coupling cell and flow submodels

**Figure 4.**
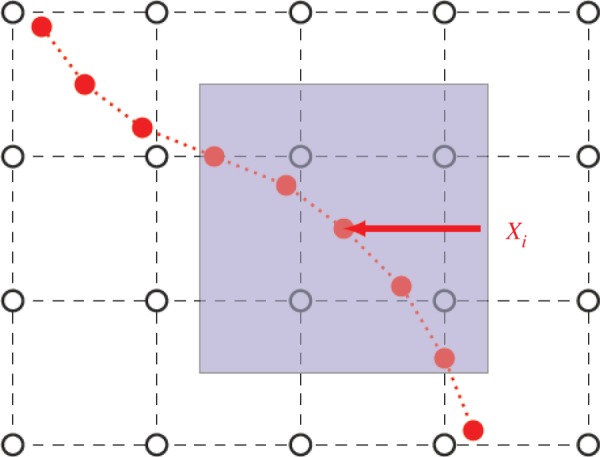
Eulerian fluid grid (black) and Lagrange solid elements (red). A Eulerian description is used for the fluid dynamics, and a Lagrangian description is used for objects immersed in the fluid. The communication between these two coordinate systems is realized by the immersed boundary method. (Online version in colour.)

To couple the integrated platelet and stochastic DAM submodels with the blood flow computed by the LBM, we use the IBM [16]. In the IBM (figure 4), the Eulerian description is used for the fluid dynamics, and the Lagrangian description is used for objects immersed in the fluid. Using lowercase letters for Eulerian variables, and uppercase letters for Lagrangian variables, we have
3.31
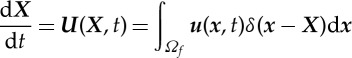

and
3.32
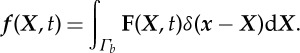

where ***t*** is time, ***u*** the flow velocity, ***U*** the speed of the solid object boundary, ***x*** the fluid flow coordinate, ***X*** the boundary coordinate, ***f*** the force density on the fluid node, ***F*** the force density on the solid elements and *δ*(***r***) the Dirac delta function.

Equations (3.31) and (3.32) are approximated using a regularized discrete delta function *δ*_*h*_. The discretized forms of equations (3.31) and (3.32) using *δ*_*h*_ are as follows
3.33
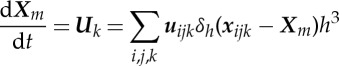

and
3.34
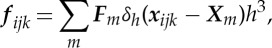

where *h* is the fluid node spacing, ***x***_*ijk*_=(*ih*,*jh*,*kh*) the coordinate of the *i*,*j*,*k*th Eulerian grid node, ***X***_*m*_ the Lagrange coordinate of the *m*th elements, ***f***_*ijk*_ the force density on ***x***_*ijk*_, ***F***_*m*_ the force density on ***X***_*m*_, ***u***_*ijk*_ the velocity of ***x***_*ijk*_, ***U***_*k*_ the velocity of **X**_*m*_. The discrete delta function *δ*_*h*_ appearing in equations (3.33) and (3.34) is a smoothed approximation to the Dirac delta function *δ*(***r***). (The detailed derivation procedures in several forms have been presented in the literature [40].) We use the following common form
3.35
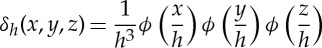

and
3.36
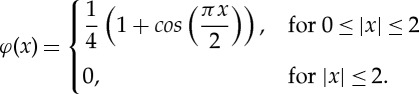



To sum up, first, in each step of the simulation, equation (3.20) is solved. Then, positions of nodes of the platelet membrane are updated by equation (3.33). Finally, the MC computations are performed to break the already formed bonds and to generate new bonds from unbound GPIb*α* and vWF.

## 4. Results

First, the model was verified by comparing simulation results with analytical solutions and available model simulation data [24]. Next, we validated the model by comparing the simulation results with the experimental data [1] on flipping platelets flowing over a vWF-coated surface. A calibrated three-dimensional model was used to predict how the stiffness of a platelet membrane, the number of receptors on a platelet membrane and the strength of platelet–platelet adhesion affect the paused time of the platelet adhering to a vessel wall.

### (a) Validation of fluid–platelet coupling by comparing with the Jeffery orbit

**Figure 5.**
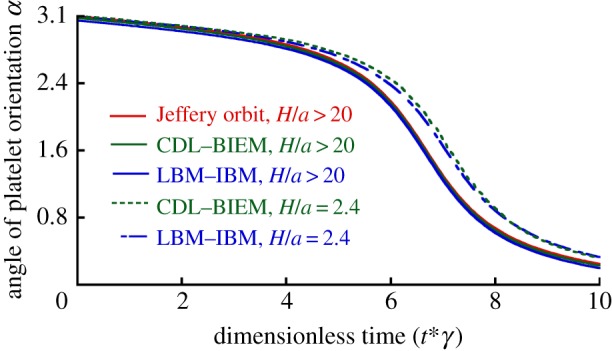
Validation of the platelet dynamics model. The analytical solution for the platelet rotational trajectory (Jeffery orbit), trajectory calculated by a completed double-layer–boundary integral equation method (CDL-BIEM) and our simulation (LBM-IBM) are shown by solid and dashed colour lines (inset key). (Online version in colour.)

**Figure 6.**
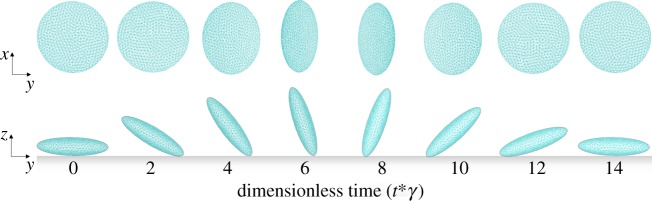
The configuration of the simulated platelets at different times flipping over the vessel wall for the wall shear stress of 3.0 dyn cm^−2^. Image sequence (*a*) shows projection of the platelet on the *x*–*y* plane from dimensionless time point 0 to 14. Image sequence (*b*) shows projection of the platelet on the *y*–*z* plane from dimensionless time point 0–14. The coordinate system is defined in figure 2*a*. (Online version in colour.)

Mody *et al.* [10] described theoretical solutions using the Jeffery orbit theory and provided predictions obtained using the analytical platelet-flipping model. This analytical solution (shown as solid red line in figure 5) did not consider the wall effect and applied only to the cases of platelet motion far from the wall (*H*/*a*>20) [24], where *H* is the centroid height of platelet and *a* is the major radius (as shown in figure 2). Mody *et al.* [24] modified the completed double-layer-boundary integral equation method to include a flat surface boundary that was used to compute the effects of the wall on the flow behaviour of a platelet. Platelets located as far as 2.4-fold platelet radii from the surface ‘display modified’ Jeffery orbits with periodic rotational motion in the direction of flow (green dashed line in figure 5). To verify our model, we simulated the flipping of a single platelet located at the distance of 2.4*a* as well as greater than 20*a*, from the vessel wall. Our simulations revealed that the calculated orbit of rotation (blue dashed line in figure 5) agreed well with the Mody's simulation results [24] (green dashed line) within an experimental error of 2.65% for platelet located at the distance of 2.4*a*, and agreed perfectly with Mody's simulation results [24] for platelets located at a distance of more than 20*a*. Figure 6 shows the series of snapshots from our simulations of platelet-flipping in a shear flow near the vessel wall. In our model, the platelet was modelled as an elastic cell with the elastic modulus measured by the AFM experiments [11], whereas Mody *et al.* [10] considered the platelet as a rigid object. By comparing our simulation results and results of Mody *et al.* [10], we conclude that our simulations can be successfully implemented to model the motion of individual resting platelets revealing high stiffness membrane values.

### (b) Validation of the model of the platelet–substrate adhesion

To validate the kinetic submodel, we simulated flowing platelets adhering to substrate through GPIb*α*–vWF binding and calculated *k*_off_ rates to compare with available experimental data. The model parameters used in our simulations (table 2) were obtained in biological experiments [1,11,22–25,27]. The adhesive dynamic parameters were measured in *in vitro* flow chamber tests [1].

**Figure 7.**
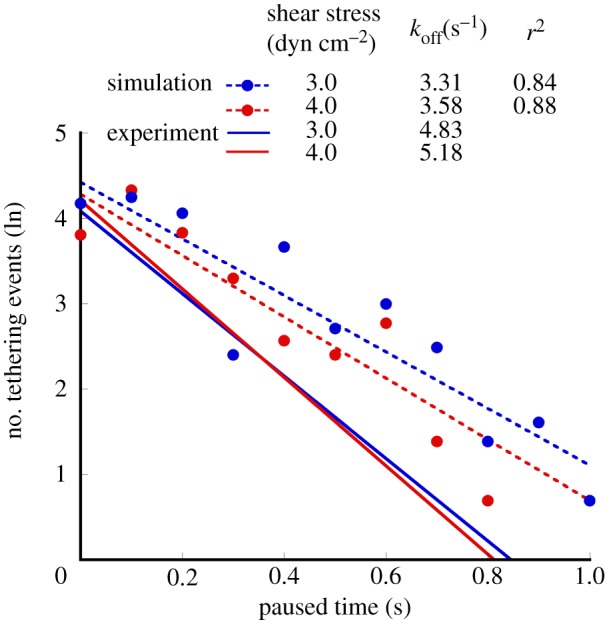
The number of tethering events as a function of the platelet paused time. The solid lines are the fitting lines of experimental data for shear stresses of 3.0 dyn cm^−2^ (shown in blue) and 4.0 dyn cm^−2^ (shown in red). The corresponding slopes of the fits (*k*_off_ values) are −4.83 and −5.18. The dashed lines are the fitting lines of simulation results (shown with circles) for shear stresses of 3.0 (shown in blue) and 4.0 dyn cm^−2^. The corresponding slopes of the fits (*k*_off_ values) are −3.31 and −3.58. (Online version in colour.)

Doggett *et al*. [1] measured the kinetics that governs platelet interactions with vWF in haemodynamic flow. In their experiment, the frequency of tethering for platelets was measured by determining the percentage of cells that paused, but did not translocate, on vWF substrates. The frequency of tethering for microspheres coated with vWF on antibody-immobilized platelet substrates was also measured. A transient tether event was defined as flowing platelets that abruptly halted forward motion for a defined period of time and subsequently released, without evidence of translocation, to resume a velocity equivalent to that of a non-interacting cell. Dissociation rate constants (*k*_off_) were determined by plotting the natural log of the number of beads that interacted as a function of pause time after the initiation of tethering (figure 7, the slope of the line is −*k*_off_).

To calculate dissociation constants, we performed simulations for various numbers of random seeds (1000–1200). The results of the simulations and experimental data are presented in figure 7 for two different flow shear rates as the natural log of the number of platelet tethering events versus the pause time. The values of dissociation rate *k*_off_ were found to be 3.31 and 3.58 s^−1^ for flow shear stress 3.0 and 4.0 dyn cm^−2^, respectively. The corresponding experimental values obtained in [1] were 4.83 and 5.18 s^−1^.

It should be mentioned that our simulations confirmed several experimental observations. It was reported in [1] that in the range of flow rates considered, forces acting on the GPIb*α*–vWF bond were not sufficient to alter the rate of dissociation *k*_off_. Our simulations also demonstrated that the *k*_off_ values altered only in a small range from 3.31 to 3.58 s^−1^. Additionally, it was reported in [1] that the forces acting on a platelet in shear flow were 14.7 and 19.6 pN for flow shear stress 3.0 and 4.0 dyn cm^−2^, respectively, whereas our model yielded very close force values of 12.8 and 15.6 pN, respectively.

Our simulations also confirmed that in the range of flow rates (0–4 dyn cm^−2^ wall shear stress), bond association and dissociation kinetics can be successfully described by the Dembo model (equations (3.12) and (3.13)).

### (c) Predictive simulations

The responses of a platelet to interactions with the environment depend, among others, on the mechanical forces that platelets experience. Here, we consider effects of platelet membrane tension, flow shear stresses and adhesion bond forces on platelet–substrate adhesion dynamics.

#### (i) Effect of platelet membrane stiffness

**Figure 8.**
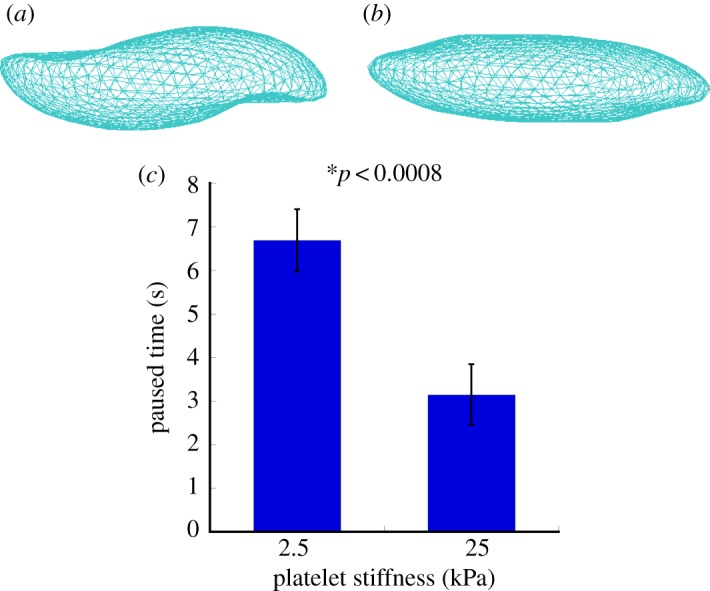
The simulated deformations of platelet structures during their adhesion to the vessel wall for platelet stiffness of 2.5 (*a*) and 25 kPa (*b*). The effect of the platelet membrane stiffness on the platelet paused time (*c*). The paused time was 6.69±0.71 s (mean±SD) for the membrane stiffness of 25 kPa, which was about twice higher than the paused time of 3.15±0.69 s (mean±SD) for the membrane stiffness of 2.5 kPa. (Online version in colour.)

Simulation results in §4*a* show that the flow dynamics of the platelet in linear shear flow can be studied by modelling platelets as rigid objects. How the stiffness of the platelets affects the platelet–substrate interaction remains to be answered. In [41], it was reported that alteration of platelet stiffness can modulate platelet aggregation. We hypothesized that softer cells lead to prolonged adhesion time and could potentially increase chances of platelets being activated after adhesion. Here, we report the simulation results indicating remarkable changes in platelet paused time as the platelet membrane stiffness changes. We varied the platelet membrane stiffness from 25 to 2.5 kPa, and performed simulations with 30 different random seeds to obtain 30 different paused times under flow shear stress of 3.0 dyn cm^−2^. The paused time was 6.69±0.71 s (mean±SD) for the membrane stiffness of 25 kPa, which is about twice higher than the paused time 3.15±0.69 s (mean±SD) for the membrane stiffness of 2.5 kPa (*t*-test, *p*<0.0008, figure 8). The total deviation of all the nodes in the deformed shape in figure 8*a* is 3.5 μm compared with the reference configuration, and in figure 8*b* is 0.28 μm. Thus, these simulation results indicated that softer cells have prolonged average paused time.

#### (ii) Effect of the number of GPIb*α* receptors expressed on the platelet membrane

**Figure 9.**
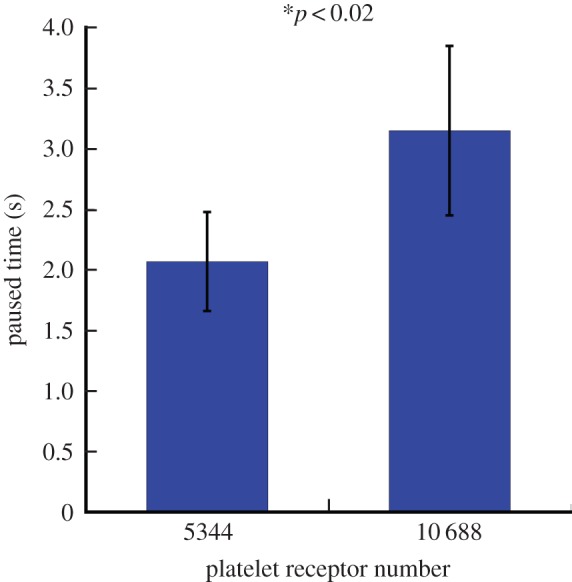
The effect of the number of platelet receptors on the platelet–vessel wall paused time. The platelet paused time for a decreased number of GPIb functional receptors was 2.07±0.41 s (mean±SD), which was significantly lower than the paused time of platelets having the normal number of receptors (3.15± 0.69 s, mean±SD). (Online version in colour.)

The interaction between platelet glycoprotein (GP) Ib–IX–V complex and vWF is the first step of the haemostatic response to vessel injury. As resting platelets interact with vWF, binding of vWF to GPIb*α* initiates platelet activation [42]. Meanwhile, in platelet-type von Willebrand disease, mutations of GPIb functional receptors can compromise haemostasis by increasing the affinity for vWF [43,44]. Some studies demonstrated that abnormalities in the concentrations of GPIb membrane proteins are present in patients with myeloproliferative disorders. In particular, decreased GPIb concentrations were found in patients with thrombocythaemia and leukaemia [45,46]. How the platelet–substrate adhesion dynamics and subsequent platelet activation are affected by the number of GPIb is not clear. The objective of our simulations performed in this section was to gain insights into this problem. We varied the platelet receptor number from 10 688 (normal) to 5344 (insufficient), and performed simulations with 30 different random seeds to obtain 30 different paused times under flow shear stress of 3.0 dyn cm^−2^. The results of our simulations revealed that the paused time in the case of decreased receptor number was 2.07±0.41 s (mean±SD), which was significantly lower than 3.15±0.69 s (mean±SD) for the normal receptor number group (*t*-test, *p*<0.02, figure 9). Our simulations predicted that as the number of GPIb on the platelet membrane decreased, the paused time of platelet adhesion to vessel wall also decreased. Thus, the results of our model suggest that the number of functional GPIb is an important factor determining platelet adhesion and subsequent activation. This has important biological consequences, as controlling the number of functional GPIb receptors can provide means for development of novel anti-thrombotic drugs. The mechanism of these drugs is based on inhibiting/promoting the function of platelet GPIb receptors to decrease/increase adhesion of platelets to vWF to control blood clot growth [47].

#### (iii) Effect of the platelet–platelet adhesion

**Figure 10.**
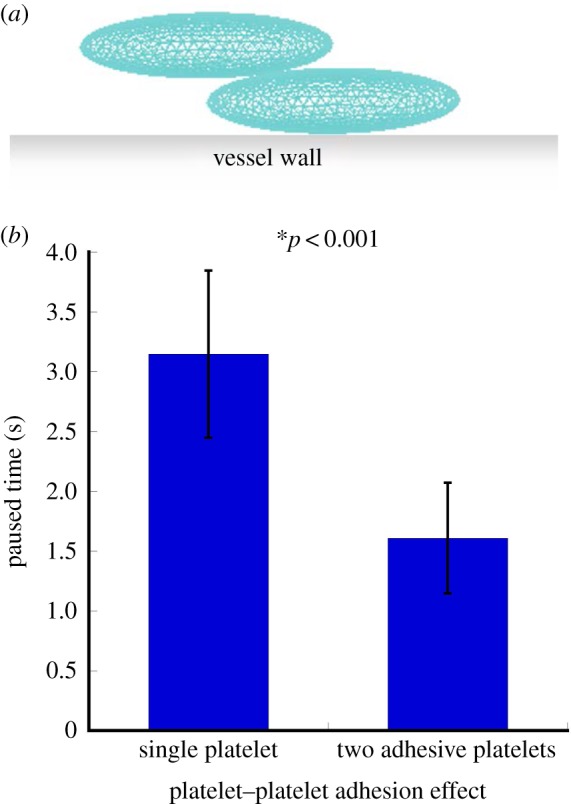
(*a*) Initial configuration of two platelets used in simulations studying the effect of mutual interaction of platelets on platelet–wall adhesion. (*b*) Platelet–vessel wall paused time as a function of the number of interacting platelets. The platelet paused time was 1.61±0.46 s (mean±SD) for two adhesive platelets, which was significantly lower than the stopping time of platelets having the normal number of receptors (3.15±0.69 s, mean±SD). (Online version in colour.)

To study how platelet–platelet interaction affects platelet adhesion to the blood vessel wall, we modelled dynamics of two platelets near the surface of the vessel (figure 10*a*). In the model, the two platelets interacted with each other and one of them adhered to the vessel wall. Our simulations revealed that the platelet paused time was 1.61±0.46 s (mean±SD) in the case of two adhesive platelets, which was significantly lower than the pause time of 3.15±0.69 s (mean±SD) calculated for a single platelet interacted with the wall (*t*-test, *p*<0.02, figure 10*b*). These results indicate an important mechanism by which a single platelet adhesion can be affected owing to interaction with neighbouring cells. These findings have direct biological consequences and help to explain how the increased platelet concentration in blood can affect platelet–wall adherence.

## 5. Discussion

This paper described a novel three-dimensional model coupling processes at three biologically important spatial scales critical for early blood clot development and uses models to provide predictive simulations. First, our model provides a comprehensive representation of mechanical properties of a platelet based on the implementation of a hybrid membrane submodel to describe mechanical behaviour of the cytoskeleton network and the lipid bilayer of the platelet. In previous studies, platelets were modelled as rigid bodies [24,33,48]. However, it has been experimentally shown [11] that platelets exhibited both elastic and viscoelastic behaviour and that they undergo large deformation in shear flow [12].

Experimental studies demonstrated [1,49,50] that flow shear stress could increase both bond formation and dissociation rates during platelet adhesion to the vessel wall. Additionally, estimates for the forces acting on platelet–substrate bonds were provided in [10]. However, Xu *et al*. [8] did not describe a detailed computational model to simulate the binding dynamics under various flow conditions. By combining three-dimensional multi-scale models with microfluidic experiments, we provided a methodology to quantify in detail single platelet-flipping in blood flow and platelet tethering to the injured vessel wall. It results in a two-way coupled fluid–cell interaction submodel combined with a stochastic submodel of formation/breakage of individual receptor–ligand bonds. This approach provided a biologically justified description of platelet dynamics, which can be used to simulate dynamics of platelets under more complex flow conditions.

By incorporating physiological parameter values characterizing cellular membrane mechanics, our method provides explicit representation for the structure of the cytoskeleton and simulation of cellular dynamics. Thus, our model allows one to examine how the mobility of cells is affected by their membrane structural and mechanical properties and, hence, aids in providing prognostic assessment in blood cell disorders outcome. The model developed in this paper can be also used for simulating important biomedical problems which involve description of dynamics and deformation of cells in fluid flow, including (patho)physiological inflammation involving leucocyte and platelet tethering to the vessel wall. Other important applications of the model include studying cell aggregate formation in blood, metastasis of tumour cells as well as stem cell attachment to the target tissues.

## Appendix A. GPU implementation

The CPU used for our simulation is the Intel Xeon CPU L5520 with clock rate 2.27 GHz. Our NVIDIA graphics card is the GeForce GTX 480, Clock rate: 1.45 GHz, CUDA driver version: 5.50, CUDA runtime version: 5.50, CUDA capability version: 2.0. GPUs are separate devices with their own processors, and memory devices which do not have direct access to the CPUs or CPU memory units. The communication pathway for transferring data between CPU memory and GPU memory has a relatively slow bandwidth capability compared with direct access to memory devices. Thus, it is necessary to minimize communication as much as possible. The typical GPU code is composed of three main parts: (i) initialization, (ii) execution, and (iii) clean-up. During initialization, model data are firstly allocated and initialized in the CPU memory. The CPU code then initializes connection to GPU device and allocates GPU memory for the model simulation data. The model data are copied from CPU memory to GPU memory units. During execution, GPU kernel functions are called and occasionally copy data between CPU and GPU memory devices. When a simulation is finished, both CPU and GPU memory units are freed and connection to GPU device is shut down for the clean-up.

Figure 1 shows the flow chart of our simulation algorithm. For a single step of the simulation, its execution on the GPU starts with the hybrid membrane model. Assuming that a platelet consists of N triangle mesh elements and P nodes (each node represents an SCE; figure 2*b*), The GPU kernel function to calculate forces in equation (3.3) has the form

sem_Force_kernel<<< blocksPerGrid, threadsPerBlock >>>(grids->devImage){

 *//Calculation of forces acting on subcellular elements*

 … …

}

where *blocksPerGrid*and *threadsPerBlock* are determined according to the block and thread distribution on the GPU card, and *P*=*blocksPerGrid*threadsPerBlock*. If this is implemented on a single CPU in serial configuration, there will be a total of *P* iterations for one step of simulation. In our GPU implementation, this simulation is performed simultaneously on *P* GPU threads. Hence, it reduces the complexity of execution time from *O*(*P*) to *O*(1) for one step of simulation. Similarly, we use the following GPU functions to calculate forces owing to bending, area constraint and volume constraint

sem_Bending_kernel<<< blocksPerGrid, threadsPerBlock >>>(grids->devImage){

 *//Calculation of forces due to bending*

 … …

}

sem_Area_Volume_kernel<<< blocksPerGrid, threadsPerBlock >>>(grids->devImage){

 *//Calculation of forces due to area and volume constraint*

 … …

}

where *blocksPerGrid* and *threadsPerBlock* are determined according to the block and thread distribution on the GPU card, and *N*=*blocksPerGrid*threadsPerBlock*. It reduces the complexity of execution time from *O*(*N*) to *O*(1) comparing with CPU code.

The forces acting on solid nodes spread to the neighbour fluid nodes using the IBM. The GPU kernel function has the form

fluid3d_force_distribute_kernel<<<blocksPerGrid3D,threadsPerBlock3D>>>(grids->devImage){

 *//Implementation of IBM*

 … …

}

where both *blocksPerGrid3D* and *threadsPerBlock3D* have three-dimensional structure similar to a three-dimensional space coordinate. Let blocks per grid 3*D*=(*x*_*b*_,*y*_*b*_,*z*_*b*_) and threads per block 3*D*=(*x*_*t*_,*y*_*t*_,*z*_*t*_), and the fluid lattice has the size of *X*×*Y* ×*Z*, then *X*=*x*_*b*_×*x*_*t*_, *Y* =*y*_*b*_×*y*_*t*_ and *Z*=*z*_*b*_×*z*_*t*_. It reduces the complexity of execution time from *O*(*XYZ*) to *O*(1) comparing with CPU code. Similar strategy is applied to lattice Boltzmann method implementation.

There are *M* receptors in each of the N triangle mesh elements of a platelet. The GPU kernel function for dynamic adhesion model has the form:

sem_platelet_wall_kernel<<<blocksPerGrid, threadsPerBlock>>>(grids->devImage){

 *//Implementation of DAM for a single receptor*

 … …

}

where *N*×*M*=blocks per grid×threads per block. It can speed up the execution time NM times compared with CPU implementation.

In conclusion, the GPU will reduce the whole simulation from *O*(*P*)+*O*(*N*)+*O*(*XY* *Z*)+*O*(*NM*)∼*O*(*XY* *Z*) to *O*(1) in time complexity. Table 3 shows real execution time of 10 000 step simulation on both CPU and GPU for three different fluid grid sizes. As table 3 shows, the execution time of GPU code is only about 1/100 of the CPU version for the fluid grid size we used in this paper. While the CPU execution time increased linearly with fluid grid size, the GPU execution time increased much slower than CPU.

**Table 3.** Execution time of 10 000 simulation steps on CPU and GPU for different fluid grid sizes.

fluid grid size	CPU (s)	GPU (s)
80×300×20	36 251	268
40×150×10	2393	231
20×75×5	232	51
